# Multi-Cell Type Glioblastoma Tumor Spheroids for Evaluating Sub-Population-Specific Drug Response

**DOI:** 10.3389/fbioe.2020.538663

**Published:** 2020-09-15

**Authors:** Hemamylammal Sivakumar, Mahesh Devarasetty, David E. Kram, Roy E. Strowd, Aleksander Skardal

**Affiliations:** ^1^Department of Biomedical Engineering, The Ohio State University, Columbus, OH, United States; ^2^The Ohio State University and Arthur G. James Comprehensive Cancer Center, Columbus, OH, United States; ^3^Wake Forest Institute for Regenerative Medicine, Wake Forest School of Medicine, Winston-Salem, NC, United States; ^4^Section of Pediatric Hematology and Oncology, Department of Pediatrics, Wake Forest Baptist Medical Center, Medical Center Boulevard, Winston-Salem, NC, United States; ^5^Comprehensive Cancer Center at Wake Forest Baptist Medical, Winston-Salem, NC, United States; ^6^Department of Neurology, Wake Forest Baptist Medical Center, Winston-Salem, NC, United States; ^7^Virginia Tech-Wake Forest School of Biomedical Engineering and Sciences, Wake Forest School of Medicine, Winston-Salem, NC, United States; ^8^Department of Cancer Biology, Wake Forest School of Medicine, Winston-Salem, NC, United States; ^9^Department of Molecular Medicine and Translational Science, Wake Forest School of Medicine, Winston-Salem, NC, United States

**Keywords:** glioblastoma, spheroid, organoid, drug response, tumor heterogeneity

## Abstract

Glioblastoma (GBM) is a lethal, incurable form of cancer in the brain. Even with maximally aggressive surgery and chemoradiotherapy, median patient survival is 14.5 months. These tumors infiltrate normal brain tissue, are surgically incurable, and universally recur. GBMs are characterized by genetic, epigenetic, and microenvironmental heterogeneity, and they evolve spontaneously over time and as a result of treatment. However, tracking such heterogeneity in real time in response to drug treatments has been impossible. Here we describe the development of an *in vitro* GBM tumor organoid model that is comprised of five distinct cellular subpopulations (4 GBM cell lines that represent GBM subpopulations and 1 astrocyte line), each fluorescently labeled with a different color. These multi-cell type GBM organoids are then embedded in a brain-like hyaluronic acid hydrogel for subsequent studies involving drug treatments and tracking of changes in relative numbers of each fluorescently unique subpopulation. This approach allows for the visual assessment of drug influence on individual subpopulations within GBM, and in future work can be expanded to supporting studies using patient tumor biospecimen-derived cells for personalized diagnostics.

## Introduction

Glioblastoma (GBM) is one of the most dangerous tumors across cancer types, and amongst brain tumors, the most lethal type of tumor which is currently incurable. Gold standard treatment includes maximally aggressive surgery to remove the bulk of the tumor and chemoradiotherapy to address remaining tumor cells. Yet despite such a comprehensive therapeutic intervention, median survival for patients is 14.5 months (Stupp et al., 2005). As these tumors progress, they infiltrate normal brain to the point where they are difficult to entirely remove surgically. Furthermore, even after what is considered successful treatment, GBM tumors universally recur. The majority of recurrence arises locally from radiation resistant cells within the initial treatment field. Upon recurrence, tumors are found to have a response rate to standard treatments of less than 5%, leading to a median survival of 8 months (Taal et al., 2014). GBM is characterized by genetic, epigenetic, and microenvironmental heterogeneity (Rybinski and Yun, 2016). These tumors evolve spontaneously, and in response to treatment, making selection of patient-specific therapies a challenge (Malkki, 2016; Wang et al., 2016). What drives changes in genetic profiles and GBM subtype composition remains largely unexplored and is not well understood (Mihai et al., 2015). Multiple genetically distinct tumoral clones exist and have been shown to be organized in spatially discrete regions within the tumor (Sottoriva et al., 2013). This crucial observation explains – at least from an evolutionary perspective – a critical mechanism of treatment failure: treatment abates sensitive subpopulations and selects for genetically resistant clones that drive treatment failure, the development of therapeutic resistance, and ultimately recurrence. Recent studies employing multiple biopsies of a single patient’s tumor have shown that multiple distinct GBM subtypes (i.e., mesenchymal, neural, etc.) exist within the same tumor (Sottoriva et al., 2013; Tang et al., 2015), that genetic heterogeneity even in early driver mutations such as TP53 occurs (Kumar et al., 2014), and that a genetically heterogeneous population of malignant cells survives initial treatment (Heimberger et al., 2003; Swartz et al., 2014).

The central challenge in conventional cancer treatment design is that there is only one reliable test bed: the patients themselves. Most often, a treatment is administered based on statistical likelihood of success in the broader population, not actual effectiveness in a particular patient. In patients with intrinsic or acquired resistance to the treatment, this results in further growth of the tumor and a loss of critical treatment time. Additional drugs can then be investigated, but only serially and with each one still being a “best guess” with diminishing probabilities of success. Moreover, during this time, due to spontaneous changes, and in response to treatments, the tumor is evolving – potentially with changes in drug responsiveness (Malkki, 2016; Wang et al., 2016). An ideal solution would be a method by which a tumor could be tracked and probed outside of the patient, where tumor evolution could be followed and multiple candidate treatments could be investigated in parallel to determine effectiveness without loss of time or potential harm to the patient. Initially, animal models seem attractive because they provide complexity reminiscent of the *in vivo* tumor physiology (Lenting et al., 2017). However, even beyond infrastructure requirements and ethical questions that accompany the use of animals, the power of these models to predict outcomes in humans is tenuous. Moreover, patient-derived xenografts (PDX) have unsatisfactory take rates, and have only been successfully established using the most malignant of tumors. *In vitro* 2D cultures have been a laboratory workhorse, but fail to recapitulate *in vivo* tissue (Baskaran et al., 2018), but 3D culture has been shown to capture a more faithful reproduction of the physiological microenvironment. In recent years, our group and others have created portfolios of tumor organoids and tumor constructs for cancer modeling and performing drug screening studies. These include cell line-based models (Skardal et al., 2015a,b, 2016; Devarasetty et al., 2017a, b; Aleman and Skardal, 2018; Shirure et al., 2018; Xue et al., 2018) as well as patient-derived tumor systems (Gao et al., 2014; Mazzocchi et al., 2018; Yan et al., 2018; Forsythe et al., 2019; Mazzocchi et al., 2019; Votanopoulos et al., 2019). However, one area that has not been addressed comprehensively is understanding and tracking which tumor subpopulations within a single tumor or tumor organoid respond or do not respond to a particular treatment. Here we describe the development and testing of a proof-of-concept GBM tumor spheroid model comprised of multiple fluorescently labeled cell lines which we use to visually track the relative contributions of each subpopulation too the overall spheroid as a result of the individual drug responses of each cell type. We demonstrate this tracking methodology using multiple GBM cell lines, but aim to deploy this approach in future work to track individual GBM subtypes within a patient-derived tumor organoid in response to therapy, thereby addressing the complexity of GBM heterogeneity.

## Materials and Methods

### Cell Culture

Four GBM cell lines were employed in several combinations in order to simulate cell population heterogeneity as one might encounter in GBM tumors. GBM cell lines U-373 MG (ATCC^®^ HTB-16^TM^) U-87 MG (ATCC^®^ HTB-14^TM^), U-87 EGFRvIII cell line (gifted by Dr. Webster Cavenee from Ludwig Cancer Research Institute, San Diego) and A172 (ATCC^®^ CRL-1620^TM^) (obtained from ATCC, Manassas, VA) were employed. All the cancer cell lines were cultured in Dulbecco’s Modified Eagle Medium (DMEM) - high glucose with 10% fetal bovine serum (FBS), 1% L-glutamine, and 1% penicillin/streptomycin in a tissue culture incubator at 37°C with 5% CO_2_. In addition, human astrocytes (Sciencell Research Laboratories, Inc., Carlsbad, CA) were cultured in Astrocyte Medium containing 2% FBS, 1% astrocyte growth supplement, and 1% penicillin/streptomycin at 37°C with 5% CO_2_. Cells were recovered from tissue culture plastic for subsequent studies using Trypsin/EDTA (Thermo Fisher, Waltham, MA).

### Cell Fluorescent Labeling

In order to allow visual tracking of cell populations within tumor spheroids, fluorescent probes were employed to label each cell line a distinct color detectable by fluorescent microscopy. Early cell-tracking studies employed 2 cell populations at a time, which were fluorescently labeled with DiI or DiO Vybrant^TM^ Multicolor Cell Labeling Kits (Thermo Fisher). Briefly, following cell recovery by Trypsin/EDTA, 5 μL of cell-labeling solution was added to 1 mL cell culture media containing approximately 1**×** 10^6^ cells and incubated for 20 min at 37°C. Cells were washed 2 times using fresh medium prior to use. Later cell-tracking studies employed 5 cell populations at a time, which were labeled with Qtracker Cell Labeling Kits (Thermo Fisher), thereby offering a wider range of wavelengths to be associated with individual cell populations. Specifically, U-87 MG cells were labeled with Qtracker 525 (green), U-87 EGFRvIII cells were labeled with Qtracker 585 (yellow), U-373 MG cells were labeled with Qtracker 705 (purple), and A172 cells were labeled with Qtracker 655 (red).

### Spherical Organoid Formation

GBM organoids were formed by self-aggregation of cells in the bottoms of non-adherent round bottom 96-well plates (Corning, Corning, NY). Single cell type, dual cell type, or 5 cell type suspensions of 10,000 cells in 100 μL volumes of media were pipetted into individual wells and allowed for form cell-cell connections over the course of 3 days. The resulting spheroids or organoids were then recovered by pipetting for subsequent studies.

### Hydrogel Preparation

Tumor organoid constructs were formed using a thiolated hyaluronic acid (HA), thiolated gelatin, and polyethylene glycol diacrylate (PEGDA)-based hydrogel system (ESI-BIO, Alameda, CA) to immobilize organoids within an extracellular matrix (ECM) with high HA content like native brain ECM. Thiolated HA and gelatin components were dissolved at 1% w/v each in water containing 0.1% w/v photoinitiator (2-Hydroxy-4-(2-hydroxyethoxy)-2-methylpropiophenone, Sigma, St. Louis, MO), and mixed with a 2% w/v linear polyethylene glycol diacrylate crosslinker (MW 3,400 Da) solution in a 2:2:1 ratio by volume. For construct formation, the hydrogel-precursor solution was used to resuspend single GBM organoids in 10 μL volumes of hydrogel. These volumes were pipetted into the wells of a sterile 48 or 96 well plates previously coated with cured polydimethylsiloxane (PDMS, used as a hydrophobic coating). The hydrogel precursor/organoid volumes were then exposed to UV light from a DYMAX 75 V.2 UV spot lamp for 1 s each. The constructs were then covered with 200 μL DMEM media with media changes performed every 3 days.

### Drug Studies

All drug compounds were purchased from Selleckchem (Houston, TX). Dacomitinib, an irreversible inhibitor of EGFR, erlotinib, a receptor tyrosine kinase inhibitor of EGFR, and NSC59984, a p53 pathway activator, were dissolved in DMEM at concentrations of 2 μM, 60 nM, and 100 μM. Two cell population organoids (A172 cell and astrocytes or U373 cells and astrocytes) were prepared as described above and treated with the drug compounds for 7 days. Five cell population organoids were prepared and maintained in culture for 7 days, after which they were treated with the drug compounds for 7 days. Organoids were assessed visually using fluorescent imaging on a TSI LCS macro-confocal microscope (Leica Microsystems, Wetzlar, Germany) to observe relative fluorescent levels associated with each starting cell population as an effect of drug treatment.

### Fluorescent Cell Quantification

Relative cell percentages of total organoids were calculated using ImageJ software (National Institutes of Health, Bethesda, MD). Individual macro-confocal fluorescent channel images were imported into ImageJ and converted to black and white binary images using the *Process : Make Binary* command, after which total number of white pixels was quantified by *Analyze : Measure* command. Subsequent percentages of each channel were then computed and graphed in Microsoft Excel or GraphPad Prism.

### Organoid Viability and Proliferation Assessment

To verify sufficient levels of cell viability in the organoids, LIVE/DEAD staining in parallel with quantification of ATP activity over time was employed. LIVE/DEAD staining (LIVE/DEAD viability/cytotoxicity kit for mammalian cells; Thermo Fisher, Waltham, MA) was performed on days 1, 4, and 7 of organoid culture. Spent medium was first aspirated from wells, after which a 100 μL volume of a PBS and DMEM mixture (1:1) containing 2 μM calcein-AM and 2 μM ethidium homodimer-1 was introduced. Constructs were incubated for 60 min, after which spent medium was again aspirated and replaced with clean PBS. Fluorescent imaging was performed using a Olympus FV3000 confocal microscope. z-Stacks (100 μm) were obtained for each construct using filters appropriate for both red and green fluorescence (594 and 488 nm, respectively) then overlaid.

In parallel, ATP activity was quantified on days 1, 4, and 7 as well. Spent media was removed from each well containing the organoids and replaced with 200 μL of CellTiter-Glo^®^ 3D Cell Viability Assay (G9681; Promega, Madison, WI) assay solution (100 μL of CellTiter-Glo^®^ 3D Reagent mixed with 100 μL DMEM). The well plate was then mixed vigorously on a plate shaker for 5 min to induce cell lysis. The plate was then allowed to incubate at room temperature for an additional 25 min to stabilize the luminescent product. The entire volume of media from each well was transferred to a corresponding well in a Costar solid white flat bottom Polystyrene 96 well Assay Plate. Luminescence was then quantified on a Varioskan Lux (Thermo Fisher) according to manufacturer’s instructions, thus quantifying activity, which correlates to the number of viable cells present. Values were then averaged amongst the different groups and graphed in Graphpad Prism.

### Statistical Analysis

Values for statistical analysis were calculated as the mean ± the standard deviation between replicates. For cell population evolution studies, an n = 4 was employed, and statistical significance between pairs of means were determined using Student’s *t*-tests with confidence intervals of 95% or *p* < 0.05 or < 0.01. For the ATP proliferation assay, an *n* = 3 was employed, and statistical significance between all three time points was determined using one way ANOVA with confidence intervals of 95%, although the *p*-value was < 0.001.

## Results and Discussion

### Overall Experimental Design

**FIGURE 1:**
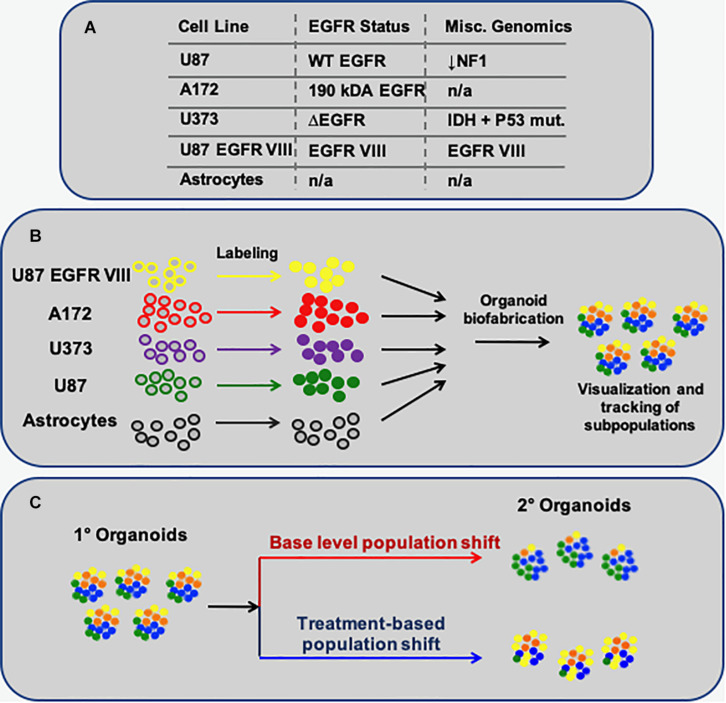
Schematic describing the methodology to model multi-subpopulation tissues/tumors as *in vitro* organoids. **(A)** The 4 GBM-derived cell lines employed, their EGFR status, and select additional miscellaneous genomic information. **(B)** Each particular cell population is fluorescently labeled, after which they are combined to form spherical organoids. **(C)** The overall concept of this approach: Following organoid formation, using fluorescent microscopy, one can visually track how each cell subpopulation – labeled with a unique fluorescent probe – changes in terms of its percent makeup of the organoids, in baseline conditions or as a response to treatments.

To begin to design a system to track GBM subtypes in 3D organoid cultures in response to chemotherapeutic treatments, we sought to employ a collection of GBM cell lines that, in terms of genomic variance, could serve as 4 distinct subpopulations GBM. These would thus act somewhat as loose representations of the 4 GBM subtypes (Verhaak et al., 2010), albeit not with the specific genomic profiles. These cell populations (Figure 1A) would then be formed into 3D spheroids and encapsulated within a hyaluronic acid-rich, and low collagen-content, hydrogel biomaterial system (Figure 1B) whose base components are commercially available (HyStem, ESI-BIO), but further customized with crosslinker molecules to drive the elastic modulus of the environment (physical stroma characteristics) toward that of brain tissue. Specifically, shear elastic modulus values, previously demonstrated (Skardal et al., 2010, 2015a,b, 2018; Sivakumar et al., 2017), fall between 200 and 1000 Pa, closely resembling the elastic of brain tissue. The overall concept of the resulting GBM organoid models would be to fluorescently label each individual cellular subpopulation with unique fluorescent probes (quantum dots), after which they would be combined into spheroids, encapsulated in the brain biomimetic ECM hydrogels, and subjected to drug screens. Subsequently, the changes in the fluorescent cellular subpopulations would be analyzed as an effect of the drug treatments (Figure 1C; Xie et al., 2015).

### Proof of Concept 2-Cell Type Spheroid Testing

**FIGURE 2:**
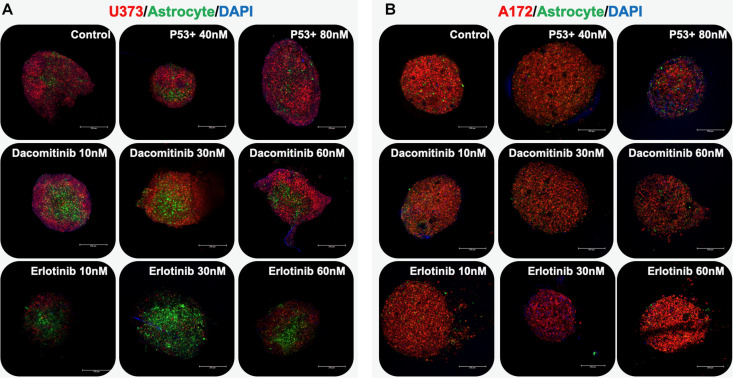
GBM organoids respond differentially based on cell line type. Organoids were comprised of a GBM cell line (red) and astrocytes (green) and nuclei (blue) imaged by macro-confocal microscopy in response to therapies. **(A)** U373-based organoids respond dramatically to erlotinib. **(B)** A172-based organoids, which have a p53 mutation, respond to a p53 activator compound to some degree. Scale bars: 250 μm.

First, a proof of concept of this methodology was tested using a 2-cell type system – glioma cells paired with astrocytes. Spheroids were formed by pipetting either U373 cells or A172 cells together with astrocytes in non-adherent, round bottom 96 well plates and allowing the cells to aggregate over the course of 2–3 days. Prior to spheroid formation, the glioma cells were labeled with a fluorescent red membrane dye (DiI, Thermo Fisher) and astrocytes were labeled with a fluorescent green membrane dye (DiO, Thermo Fisher). Following aggregation, spheroids were embedded in the HA-gelatin hydrogel and maintained until subsequent drug studies. Three compounds were employed: dacomitinib (an irreversible EGFR inhibitor), erlotinib (another EGFR inhibitor), and a P53 activator. While these compounds are not understood to be clinically actionable, they were selected to support model validation. With respect specifically to the P53 activator, P53 is the most mutated gene in GBM with an occurring rate of 84% percentage in glioblastoma patients and also 94% of GBM cell lines (Zhang et al., 2018). Using a p53 activator is a good proof of principle as it is explored as an alternative target in GBM treatment. After 7 days of drug exposure, the spheroids were stained with DAPI and imaged by macro-confocal microscopy in the red, green, and DAPI channels after which images from each channel were overlaid to form composites (Figure 2). The U373 and astrocyte spheroids show that the relative U373 cell number decreases somewhat when treated with dacomitinib, but much more substantially with erlotinib. This is evident from the larger percentage of cells that are green-labeled astrocytes that are visible compared to the untreated control spheroids (Figure 2A). This makes sense given the EGFR amplification has been associated with U373 cells. While both dacomitinib and erlotinib are EGFR inhibitors, differential responses to these drugs by U373 cells have been demonstrated previously. Specifically, in 2D cell cultures U373 cells did not respond significantly to dacomitinib, except at large doses (Zhu and Shah, 2014) while they did respond to Erlotinib through EGFR inhibition (Ramis et al., 2012). The, response to the P53 activator is less pronounced, yet there does seem to be some effect as seen from the visible astrocytes in the 40 nM-treated condition. In comparison, the A172 and astrocyte spheroids show little response to any of the drug conditions (Figure 2B).

### Four-Cell Type Spheroid Drug Response Population Tracking

To increase the complexity in order to better mimic the *in vivo* tumor heterogeneity of the system, spheroids were created using the 4 glioblastoma cell lines described in Figure 1. Specifically, the cell lines U87, U373, A172, and U87 EGFR VIII were employed to roughly simulate the 4 subtypes observed in clinical glioblastoma tumors. It should be noted that these cell lines are not complete representations of these subtypes, but can be distinguished from one another based on genetic expression profiles. The U87 cell line serves as a model for the mesenchymal subtype as it is EGFR (epidermal growth factor receptor) wild type and has decreased NF1 (neurofibromin) expression. The U373 cell line has IDH and P53 mutations, similar to the proneural subtype. The U87 EGFR VIII cell line mimics EGFR expression in the classical subtype. This leaves the A172 cell line, which has a larger molecular weight (190 kDa) EGFR, to serve as a model for the neural subtype. To be clear, the goal here is not to accurately represent each subtype, but to provide 4 distinctly different subpopulations to serve as representations of 4 potential subtypes, thus providing GBM-like heterogeneity in a new organoid model system.

**FIGURE 3:**
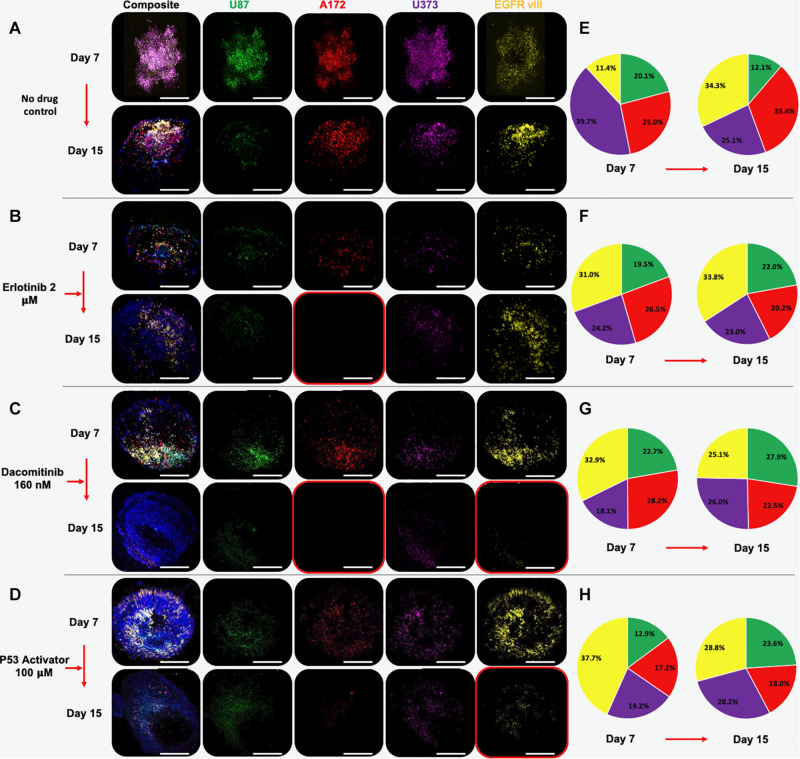
Multi-cell type GBM organoids experience subpopulation shifts in response to therapeutic interventions. Organoids are comprised of U87 (green), A172 (red), U373 (purple), and U87 EGFR VIII (yellow) cells. Panels show composite and unmixed fluorescent channels at days 7 and 15. **(A)** Under no drug control condition, all cell types remain by day 15, although A172, U373, and U87 EGFR VIII cell populations outweigh U87 cells. **(B)** Erlotinib causes a significant decrease in the U87 and A172 population, while the EGFR vIII positive population appears to increase in number. **(C)** Dacomitinib causes decrease in A173 and EGFR VII populations. **(D)** A P53 activator causes decrease in A173 and EGFR VII populations. Images taken at day 7 and after a 7-day drug treatment on day 15. **(E–H)** Color-matched pie charts in which relative percentages of each GBM cell line are shown at days 7 and 15 for each condition.

GBM spheroids were created in the same manner as described above, only using all 4 GBM cell lines. Prior to spheroid formation, each cell line was fluorescently labeled using Qtracker probes. Specifically, U-87 MG cells were labeled with Qtracker 525 (green), U-87 EGFRvIII cells were labeled with Qtracker 585 (yellow), U-373 MG cells were labeled with Qtracker 605 (orange), and A172 cells were labeled with Qtracker 655 (red). After 7 days in hydrogel culture, the spheroids were subjected to 7-day drug screens using the 3 compounds described above. Before and after drug exposure, macro-confocal microscopy was used to capture each cell population’s fluorescent signature and a composite image (Figure 3).

**FIGURE 4:**
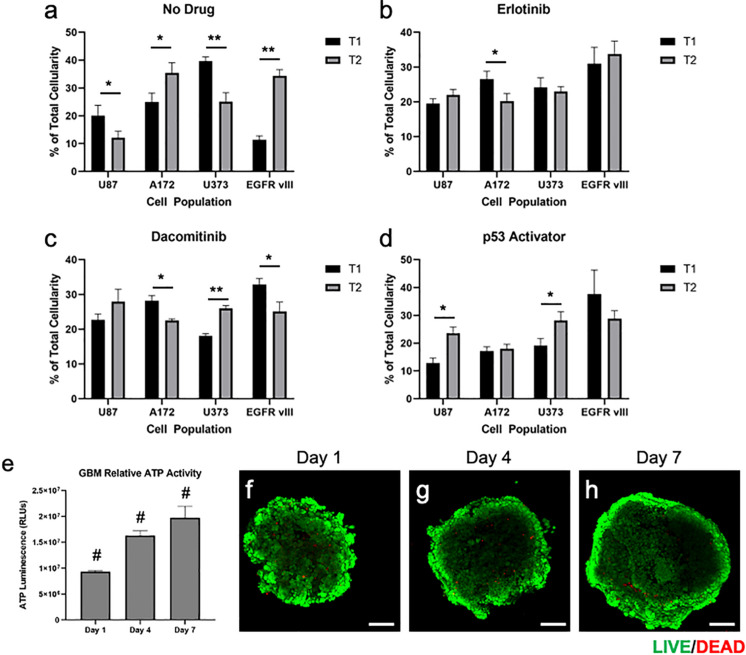
Quantification and statistical analysis of multi-cell type GBM organoids in response treatments and baseline viability validation. **(a–d)** Percentage of total cellularity for each cell type is shown at T1 (7 days) and T2 (15 days) for **(a)** no drug, **(b)** erlotinib, **(c)** dacomitinib, and **(d)** the p53 activator. Drugs were administered at day 7 following the documentation at the first time point. Statistical significance: *p < 0.05; **p < 0.01. **(e–h)** Quantification of ATP activity over time and visualization of GBM organoid baseline viability. Multi-cell type GBM organoids were maintained in culture for 7 days following formation, during which **(e)** ATP was quantified on days 1, 4, and 7, showing increasing ATP activity over time. **(f–h)** In parallel, LIVE/DEAD staining was performed on days 1, 4, and 7, after which organoids were imaged by fluorescent confocal microscopy. Scale bars – 100 μm; Green calcein AM-stained cells – viable cells; Red ethidium homodimer-stained cells – dead cells. Statistical significance: ^#^*p* < 0.001 between the 3 time points.

Multi-cell type GBM spheroids that were not subjected to drugs (i.e., no drug control) were observed to undergo some rearrangement, or self-organization of cell populations. In addition, it appeared that the U87 and U373 cell populations did not maintain their relative presences in the spheroids compared to the other 2 cell populations, which actually increased in terms of relative percentage (Figures 3A, 4a). Understanding that this model is built on cell lines, this is a scenario that occurs in patients. Some glioma cell subpopulations excel, while other do not (Xie et al., 2015; Lloyd et al., 2016). This intra-cell type heterogeneity is a feature that few models have yet to include (Caragher et al., 2019).

However, when drug screens were performed, sub-population ratios clearly shifted visually in other ways. Erlotinib treatment resulted in significant decreases in A172 cell populations (Figures 3B, 4b). This is in comparison to treatment with dacomitinib, which appears to have had statistically significant cell killing effects in the A172 and U87 EGFR VIII cell populations, while the average U373 relative percentage increased (Figures 3C, 4c). First generation tyrosine kinase inhibitors (TKI) like erlotinib are not effective against EGFR mutations observed in glioblastoma. Specifically, EGFR VIII is a specific resistance mechanism (Schulte et al., 2013). Second generation TKI like dacomitinib are used when there is an observed resistance to first generation TKI drugs (Chiba et al., 2017). This phenomenon was observed in our model. Lastly, as described in above studies, an experimental P53 activator compound was tested. The treatment of this compound resulted in a decrease in U87 EGFR VIII cell populations, although this result was not significant due to a large standard deviation at timepoint 1. Conversely, the U87 and U373 cell populations increased in relative percentage (Figures 3D, 4d). We then included quantification of the relative percentage of each GBM subpopulation at each time point for the no drug group and each drug treatment. Figures 3E–H show these relative percentages via pie charts, allowing straightforward depiction of the evolution or varied subpopulation response to their environmental conditions.

### Confirmation of Cell Viability and Proliferative Capacity in Organoids

While the focus of our studies was specifically on the relative contributions of each cell population to the overall organoid, rather than cell viability, we chose to also verify that the organoids maintained high levels of viability while in culture. This was performed by performing a series of ATP activity quantification assays and LIVE/DEAD staining and imaging over time in culture. Measurements of ATP activity showed that overall relative activity in the GBM organoids increased from days 1 to 4 and from days 4 to 7, ndicating overall positive proliferation of cells within the organoid over time (Figure 4e). In addition, following LIVE/DEAD staining of GBM organoids at days 1, 4, and 7, relative viability was visualized by fluorescent confocal microscopy. As can be seen in Figures 4f–h, overall viability is high, with ethidium homodimer-stained red fluorescing dead cells only making up a small percentage of the organoids. Instead, calcein AM-stained green fluorescing viable cells make up the bulk of the organoids. It should be noted here that in Figures 4f–h, the centers of the organoid images are lower in terms of fluorescent intensity. This is an artifact that we have observed in many spheroid models, as the ability of the microscope laser to penetrate the central region of a spheroid can be limited. Lastly, a general trend of increasing size can be observed, supporting the ATP-based proliferation data. Together, these data indicate that the majority of cells in the GBM organoids are highly viable prior to any drug studies.

## Conclusion

Intratumoral heterogeneity in GBM, which is present at diagnosis, is dynamic as a consequence of both time and treatments, and is often correlated with the development of treatment resistance and disease progression, has proven to be a significant challenge in the efforts to cure this deadly disease (Sottoriva et al., 2013; Bastien et al., 2015). A reliable model that accounts for intratumoral complexity and heterogeneity, which can also be serially monitored through time and in response to various therapies, is desperately needed. Here we have described an initial attempt at such a serially accessible model using a collection of cell lines. Serial patient biopsies to monitor GBM intratumoral dynamics are not feasible; however, our model is an important first step in bridging the gap between static and complex dynamic GBM models that can better study phenotypic plasticity. In our work, we have been able to build models that capture the established GBM intratumoral heterogeneity as well as observe clonal evolution in response to time and targeted small molecule inhibitors and activators. These novel models are full of promise.

These early generation models, however, have several important limitations. The organoid cultures themselves admittedly vary in size. We believe that while the organoids begin a consistent size upon formation in the round bottom wells, after they are encapsulated in the hydrogel, there are no longer restrictions to cell migration. The cells can, but do not always, migrate outwards as the overall number of cells increases during proliferation in culture. This results in variability both in terms of size and geometry. Additionally, while we are using multiple GBM cell lines, we did not include many other features of the brain microenvironment beyond a hyaluronic acid-based ECM. However, we are continuing to work to optimize our GBM models, addressing critical components of the macroenvironment, including blood-brain-barrier drug transport and cell trafficking considerations, as well as the microenvironment, including components of the immune system and other cells found locally such as neurons and pericytes. On the successes of early studies in our lab such as these, we hope to develop accurate and reliable models of brain tumors, including patient tumor biospecimen-derived models of GBM in which each specific GBM subtype can be tracked as we describe herein, that will both inform tumor clonal evolutionary biology as well as provide tumor analogs for *ex vivo* patient-specific drug screening.

## Data Availability Statement

The datasets generated for this study are available on request to the corresponding author.

## Author Contributions

HS, RS, and AS conceived of the scientific concept. HS and AS planned the individual experiments. HS performed the majority of the experiments, while MD oversaw and performed some of the macro-confocal imaging. HS, MD, DK, RS, and AS wrote and edited the manuscript. All authors contributed to the article and approved the submitted version.

## Conflict of Interest

The authors declare that the research was conducted in the absence of any commercial or financial relationships that could be construed as a potential conflict of interest.
